# LUCAS Versus Manual Chest Compression During Ambulance Transport: A Hemodynamic Study in a Porcine Model of Cardiac Arrest

**DOI:** 10.1161/JAHA.118.011189

**Published:** 2018-12-28

**Authors:** Aurora Magliocca, Davide Olivari, Daria De Giorgio, Davide Zani, Martina Manfredi, Antonio Boccardo, Alberto Cucino, Giulia Sala, Giovanni Babini, Laura Ruggeri, Deborah Novelli, Markus B Skrifvars, Bjarne Madsen Hardig, Davide Pravettoni, Lidia Staszewsky, Roberto Latini, Angelo Belloli, Giuseppe Ristagno

**Affiliations:** ^1^ Department of Cardiovascular Research Istituto di Ricerche Farmacologiche Mario Negri IRCCS Milan Italy; ^2^ DIMET School of Medicine University of Milano‐Bicocca Monza Italy; ^3^ DIMEVET University of Milan Lodi Italy; ^4^ Dipartimento di Fisiopatologia Medico‐Chirurgica e dei Trapianti University of Milan Milano Italy; ^5^ Emergency Care and Services Department of Emergency Medicine University of Helsinki and Helsinki University Hospital Helsinki Finland; ^6^ Clinical Department Stryker/Jolife AB Lund Sweden

**Keywords:** ambulance transport, cardiac arrest, cardiopulmonary resuscitation, chest compression resuscitation, manual cardiopulmonary resuscitation, mechanical cardiopulmonary resuscitation, Cardiopulmonary Resuscitation and Emergency Cardiac Care, Translational Studies, Cardiopulmonary Arrest

## Abstract

**Background:**

Mechanical chest compression (CC) is currently suggested to deliver sustained high‐quality CC in a moving ambulance. This study compared the hemodynamic support provided by a mechanical piston device or manual CC during ambulance transport in a porcine model of cardiopulmonary resuscitation.

**Methods and Results:**

In a simulated urban ambulance transport, 16 pigs in cardiac arrest were randomized to 18 minutes of mechanical CC with the LUCAS (n=8) or manual CC (n=8). ECG, arterial and right atrial pressure, together with end‐tidal CO_2_ and transthoracic impedance curve were continuously recorded. Arterial lactate was assessed during cardiopulmonary resuscitation and after resuscitation. During the initial 3 minutes of cardiopulmonary resuscitation, the ambulance was stationary, while then proceeded along a predefined itinerary. When the ambulance was stationary, CC‐generated hemodynamics were equivalent in the 2 groups. However, during ambulance transport, arterial and coronary perfusion pressure, and end‐tidal CO_2_ were significantly higher with mechanical CC compared with manual CC (coronary perfusion pressure: 43±4 versus 18±4 mmHg; end‐tidal CO_2_: 31±2 versus 19±2 mmHg, *P*<0.01 at 18 minutes). During cardiopulmonary resuscitation, arterial lactate was lower with mechanical CC compared with manual CC (6.6±0.4 versus 8.2±0.5 mmol/L, *P*<0.01). During transport, mechanical CC showed greater constancy compared with the manual CC, as represented by a higher CC fraction and a lower transthoracic impedance curve variability (*P*<0.01). All animals in the mechanical CC group and 6 (75%) in the manual one were successfully resuscitated.

**Conclusions:**

This model adds evidence in favor of the use of mechanical devices to provide ongoing high‐quality CC and tissue perfusion during ambulance transport.

**Clinical Perspective**

**What Is New?**

- The suggestion to use a mechanical compressor to deliver sustained high‐quality chest compression (CC) in a moving ambulance has been supported only by data on the quality of cardiopulmonary resuscitation (CPR) metrics.
- This is the first investigation reporting greater hemodynamic support and systemic perfusion generated by mechanical CC compared with manual CC during ambulance transport in a porcine model of CPR. Mechanical CC accounted also for a better CC quality, with a lesser rescuer's physical effort requirements, compared with manual CC.

**What Are the Clinical Implications?**

- The study provides evidence to the current knowledge gap on mechanical CPR during transport as claimed in the 2015 International Consensus on Cardiopulmonary Resuscitation and Emergency Cardiovascular Care Science with Treatment Recommendations.
- The study may have a potential impact on the rescuers’ decision on whether stay on the scene or transport the cardiac arrest patient to the hospital with an ongoing mechanical CPR.
- Indeed, the study results encourage the use of mechanical CPR devices during ambulance transport to assure ongoing high‐quality CC, adequate hemodynamic support and tissue perfusion, and rescuers’ safety.

## Introduction

Out‐of‐hospital cardiac arrest is a leading cause of death worldwide.^1^, ^2^ Despite major efforts to improve outcome, the most recent trials have provided dismal end results with only 3% to 10% of patients surviving to hospital discharge.^3^, ^4^, ^5^, ^6^ Accordingly, prompt cardiopulmonary resuscitation (CPR) is the major determinant of successful resuscitation,^2^, ^7^ but its quality heterogeneity may contribute to the variable survival rates reported in different regions.^8^, ^9^

During CPR, provision of high‐quality chest compression (CC) may re‐establish systemic blood flow, achieving and maintaining threshold levels of coronary and cerebral perfusion.^2^, ^10^, ^11^ Nevertheless, ineffective and frequently interrupted manual CC is often provided even by well‐trained rescuers, leading to unsuccessful resuscitative efforts.^12^, ^13^, ^14^, ^15^ The challenge is even greater during transport, a condition characterized by the presence of acceleration, deceleration, and rotational forces that may affect the rescuers’ performance.^16^, ^17^, ^18^, ^19^ Thus, high‐quality manual CC in the moving ambulance is physically demanding and impractical, and might compromise providers’ safety.^16^, ^20^ For this special circumstance, the use of a mechanical CPR device, capable to deliver CC consistently, has been suggested as a reasonable alternative to manual CC.^16^

However, the above suggestion has been supported only by manikin studies or clinical data on the quality of CPR metrics.^18^, ^19^, ^21^, ^22^, ^23^, ^24^ Indeed, whether mechanical CPR is superior to manual CPR in special situations, such as the moving ambulance, has been highlighted as a knowledge gap in the 2015 International Consensus on Cardiopulmonary Resuscitation and Emergency Cardiovascular Care Science, underlying the urgency to focus research efforts on this field.^16^

This experimental study, therefore, sought to directly investigate the hemodynamic support generated by a mechanical piston device or manual CC in a moving ambulance. The hypothesis on whether mechanical CC would improve systemic perfusion compared with manual CC was tested in a preclinical porcine model of out‐of‐hospital cardiac arrest. The primary aim of the study was to assess if mechanical CC would provide a higher coronary perfusion pressure (CPP) compared with manual CC during ambulance transport. The secondary aim was the comparison of CC quality between the 2 CPR strategies during transport.

## Methods

All procedures involving animals and their care were in conformity with national and international laws and policies (Art. 31, D. Lgs n° 26/2014). Approval of the study was obtained by the institutional review board committee and governmental institution (Ministry of Health approval no. 979/2017‐PR). The data that support the findings of this study are available from the corresponding author upon reasonable request.

### Animal Preparation

Sixteen male domestic swine (34±0.5 kg) were fasted the night before the experiments except for free water access. Anesthesia was induced by intramuscular injection of ketamine (20 mg/kg) followed by intravenous administration of propofol (2 mg/kg) and sufentanyl (0.3 μg/kg) through an ear vein access. Anesthesia was maintained with a continuous intravenous infusion of propofol (4‐8 mg/kg per hour) and sufentanyl (0.3 μg/kg per hour). A cuffed tracheal tube was placed, and animals were mechanically ventilated (Bellavista 1000, IMT Medical, Switzerland) with a tidal volume of 15 mL/kg, a fraction of inspired oxygen (FiO_2_) of 0.21, and a positive‐end expiratory pressure of 5 cmH_2_O. Respiratory frequency was adjusted to maintain the end‐tidal partial pressure of carbon dioxide (EtCO_2_) between 35 and 40 mmHg, monitored with an infrared capnometer (LIFEPAK 15 monitor/defibrillator, Physio‐Control, WA).^25^

For measurement of aortic pressure, a fluid‐filled 7F catheter was advanced from the right femoral artery into the thoracic aorta. For measurements of right atrial pressure, another fluid‐filled 7F catheter was advanced from the right femoral vein into the right atrium. Conventional pressure transducers were used and connected to the monitor defibrillator (LIFEPAK 15).^25^ For inducing ventricular fibrillation (VF), a 5F pacing catheter was advanced from the right subclavian vein into the right ventricle.^26^ The position of all catheters was confirmed by characteristic pressure morphology and/or fluoroscopy. Frontal plane ECG was recorded.

## Experimental Procedure

**Figure 1:**
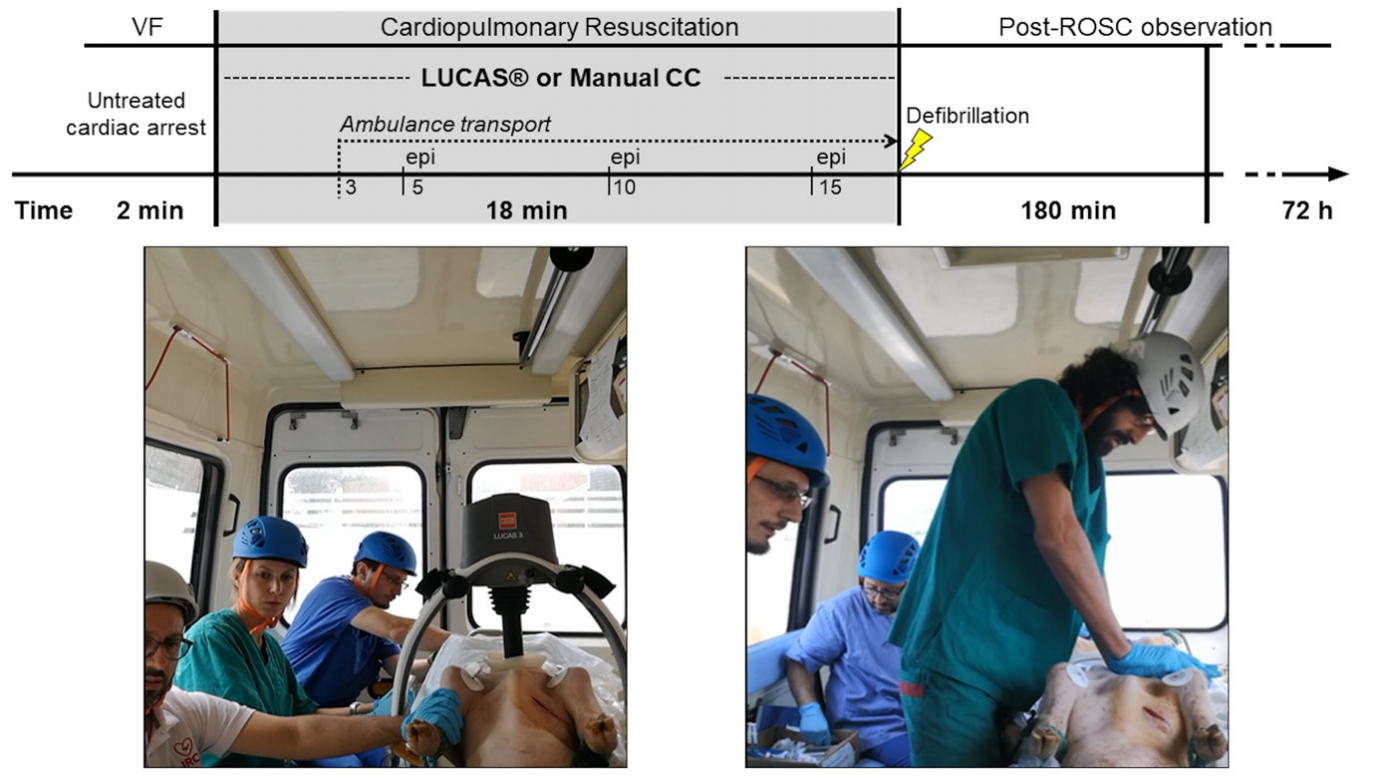
On the top: a flowchart of the study protocol. On the bottom: a view of the ambulance cabin with an ongoing mechanical chest compression on the left and manual CC on right. CC indicates chest compression; epi, epinephrine administration; ROSC, return of spontaneous circulation; VF, ventricular fibrillation.

Before inducing cardiac arrest, animals were randomized by the sealed envelope method to receive either mechanical or manual CC. Animals were then placed in a standard clinical ambulance, in use at the veterinarian hospital where the experiments were performed. Baseline measurements were obtained, and VF was electrically induced with 1 to 2 mA alternating current delivered to the endocardium of the right ventricle.^26^ Mechanical ventilation was discontinued after onset of VF. After 2 minutes of untreated VF, continuous CC with 1 of the 2 strategies, mechanical or manual, was begun and performed for 18 minutes. Mechanical ventilation with a FiO_2_ of 1.0 (Oxylog, Dräger, Lübeck, Germany) was resumed simultaneously to CC (tidal volume of 500 mL, respiratory rate of 10 breaths/min).^25^ Every 5 minutes during CPR, epinephrine (1 mg) was administered via the right atrium, while arterial blood samples were obtained to assess lactate levels. The experimental protocol is summarized in Figure 1.

During the initial 3 minutes of CPR, the ambulance was stationary, and this allowed for a comparison of CC quality and hemodynamics during a basal static condition. In the following 15 minutes, the ambulance proceeded along a predefined itinerary inside the veterinarian university campus and in the surrounding area, simulating a typical urban transportation. The route of the ambulance journey, together with the total distance traveled and the average speed, were recorded using a GPS‐based tracker app (Endomondo Sports Tracker, vers. 18.6.2). Manual CC was provided in accordance to 2015 international CPR guidelines.^2^ A group composed by the same 4 qualified CPR providers was available for all experiments. During CPR, the rescuers could see the physiologic parameters on the defibrillator monitors, ie, arterial pressure, right atrial pressure, EtCO_2_, and were allowed to optimize the CPR quality accordingly, while CC rate was guided by the monitor/defibrillator metronome. Mechanical CC was delivered by the LUCAS 3.0 chest compression system (Stryker/Jolife AB, Lund, Physio‐Control, Sweden), which delivers continuous CC (rate: 102±2 per minute; depth: 53±2 mm; duty cycle: 50±5%). The mechanical compressor was already positioned on the animal chest before inducing VF.

Beside the driver and a copilot, the ambulance cabin crew consisted of 2 certified professional rescuers who alternated each other in performing manual CC every 2 minutes, and 1 operator responsible for drug administration and arterial blood sampling. A fourth investigator, seating at the head site, provided continuous timing information to the rescuers and assured compliance to the experimental protocol, without any direct intervention in the resuscitative maneuvers.

After the 18‐minute interval of CPR, defibrillation was attempted with a single biphasic 200‐joule shock, using a LIFEPAK 15 monitor/defibrillator. Return of spontaneous circulation (ROSC) was defined as the presence of sinus rhythm with a mean arterial pressure of >60 mmHg. If ROSC was not achieved, CPR was resumed and continued for 1 minute before a subsequent defibrillation with an escalating energy strategy (300‐360‐j). If VF reoccurred after ROSC, an immediate defibrillation was delivered. The same resuscitation protocol was continued until successful resuscitation or for a maximum of 5 additional minutes.

At the end of the resuscitation maneuvers, a chest computerized tomography was performed with a 16‐slices computerized tomography scanner (GE Brightspeed, GE Healthcare, Italy) to evaluate rib fractures and other major CPR‐related injuries. Animals were then returned in the operating room, where they were monitored for an additional 3 hours, under anesthesia. Catheters were then removed, wounds were repaired, and the animals were extubated and returned to their cages. Analgesia with butorphanol (0.1 mg/kg) was administered by intramuscular injection. At the end of the 72‐hour post resuscitation observation, animals were reanesthetized for echocardiographic examination and blood sample withdrawn. Animals were then euthanized painlessly with an intravenous injection of 150 mg/kg sodium thiopental.

### Measurements

ECG, hemodynamics (arterial and right atrial pressures), EtCO_2_, and esophageal temperature were continuously recorded with 2 LIFEPAK 15 monitor/defibrillators. All data were then stored on CODE‐STAT 9.0 (Physio‐Control, WA) and subsequently exported as comma separated values (.csv) to LabChart 8.0 (ADInstruments, UK) for the analysis. The coronary perfusion pressure (CPP) was computed from the differences in time‐coincident diastolic aortic pressure and right atrial pressure.^25^, ^26^

Transthoracic echocardiography was performed using a phase‐array multifrequency 2.5‐ to 5‐MHz probe (CX50, Philips, The Netherlands). Two‐dimensional apical 4 chamber view was acquired to determine left ventricular volumes and ejection fraction calculations were computed using the modified single‐plane Simpson's rule.^25^ Cardiac output (CO) was determined as the product of the time‐velocity integral of the outflow curves (VTI) obtained in 5‐chamber apical view using pulsed wave Doppler, the cross sectional area of the left ventricular outflow tract (LVOT) obtained from 2‐dimensional echocardiography image in parasternal long‐axis view and heart rate (HR) [CO=VTI×LVOT×HR].

CC rate, CCs delivered per minute, and CC fraction (CCF) were calculated using the CODE‐STAT 9.0 CPR quality assessment tool, which uses the information derived from the transthoracic impedance (TTI). CC quality was additionally evaluated measuring the total power from the power spectral density analysis of the TTI curve after Fast Fourier Transformation (LabChart 8.0, ADInstrumets, UK).^27^ This served as a measure of variability in CC consistency (CC‐generated thoracic impedance (Impcc) variability).

CC providers’ fatigue at the end of the resuscitative maneuvers and their perception on the feasibility of CC provision during ambulance transport, intended as practicability and safety of the intervention, were evaluated using a score on a 10‐point scale from 0 (no fatigue or intervention 100% feasible) to 10 (maximal physical effort or intervention 100% impractical).

Arterial blood gases were assessed with i‐STAT System (Abbott Laboratories, Princeton, NJ). Plasma high‐sensitivity cardiac troponin T and serum neuron‐specific enolase (NSE) were measured with electrochemiluminescence assays (Roche Diagnostics, Italy).^25^

Functional recovery was evaluated before euthanasia according to overall performance categories as follows: 1=normal; 2=slight disability; 3=severe disability; 4=coma; and 5=brain death or death.^25^ Scores were assessed by veterinarian doctors masked to group treatment.

### Statistical Analysis

Shapiro–Wilk test was used to confirm normal distribution of the data. Continuous variables are reported as mean±SEM or median with interquartiles [Q1–Q3], as appropriate. Categorical variables were described as count and proportion (%). For comparisons between time‐based measurements within the 2 groups, repeated‐measures analysis of variance was used. In the case of a significant test result, a post‐hoc analysis was performed using the Fisher Least Significant Difference (LSD) test. For comparisons between groups at the given time points, 1‐way analysis of variance was used. Non‐parametric Mann‐Whitney *U* test was used for variables not normally distributed. When the dependent variable was categorical, a Fisher exact test was used. A *P*<0.05 (2‐tailed) was regarded as statistically significant. GraphPad Prism 7.0 (GraphPad Software Inc., La Jolla, CA) was used for statistical analyses.

The sample size was estimated on the mean CPP. Using CPP values from a previous study,^25^ (38.5±13.7 mmHg after 5 minutes of mechanical CC), and assuming a 50% reduction in the manual CC group during transport compared with the mechanical CC, 8 animals per group would be needed to have a power=0.8 (α=0.05, 2‐sided).

## Results

No significant differences in body weight, hemodynamics, EtCO_2_, cardiac function, arterial blood gases, and temperature were observed between the 2 groups at baseline (Table 1). No differences in the total distance traveled and average speed were noted between the 2 groups, as detailed in Table 2.

**Table 1:** Baseline Characteristics

	LUCAS (n=8)	Manual (n=8)
Body weight, Kg	35±1	34±1
Heart rate, bpm	79±6	83±8
Systolic arterial pressure, mmHg	123±5	118±8
Diastolic arterial pressure, mmHg	85±6	84±4
Right atrial pressure, mmHg	5±1	5±1
End‐tidal CO_2_, mmHg	36±1	37±1
pH	7.44±0.02	7.44±0.01
Arterial oxygen partial pressure, mmHg	86±4	80±3
Arterial carbon dioxide partial pressure, mmHg	36±1	37±1
Arterial oxygen saturation, %	97±1	96±1
Arterial bicarbonate, mmol/L	25±2	25±1
Arterial base excess, mmol/L	1±2	1±1
Left ventricular ejection fraction, %	67±3	69±4
Left ventricular end‐diastolic volume, mL	30±3	27±2
Left ventricular end‐systolic volume, mL	10±1	8±1
hs‐cTnT, pg/mL	6 [3–8]	8 [6–9]
Temperature, °C	36.7±0.3	37.2±0.2

Data are reported in mean±SEM, except for hs‐cTnT and NSE that are expressed as median [interquartile range]. hs‐cTnT indicates highsensitivity cardiac troponin T; NSE, neuron‐specific enolase.

**Table 2:** Ambulance Itinerary and Cardiopulmonary Resuscitation Quality

	LUCAS CC (n=8)	Manual CC (n=8)
Transport distance, km	7.8±0.4	8.6±0.5
Ambulance average speed, km/h	26.0±2	28.5±2
CC rate, n		
Total CPR duration	102 [102–102]	103 [101– 104]
Static	102 [102–102]*	101 [100–101]
Transport	102 [102–102]†	103 [102–105]
CC delivered per min, n		
Total CPR duration	101 [100–102]†	97 [93–99]
Static	97 [96–102]	100 [99–101]
Transport	102 [100–102]†	97 [92–99]
CCF, %		
Total CPR duration	99 [98–100]	98 [98–99]
Static	96 [95–100]†	100 [100–100]
Transport	100 [99–100]†	98 [97–99]
Imp_cc_ variability, ms^2^	2854 [1035–4584]*	16 068 [13 240–19 446]
Fatigue, score	1.2±0.3*	8.8±0.3
Feasibility, score	9.1±0.3*	3.7±0.6

Data are reported as mean±SEM or median [interquartile range]. CC indicates chest compression; CCF, chest compression fraction; CPR, cardiopulmonary resuscitation; Imp, impedance.

*P<0.01 vs manual; ^†^ *P*<0.05.

### Hemodynamics During CPR

**Figure 2:**
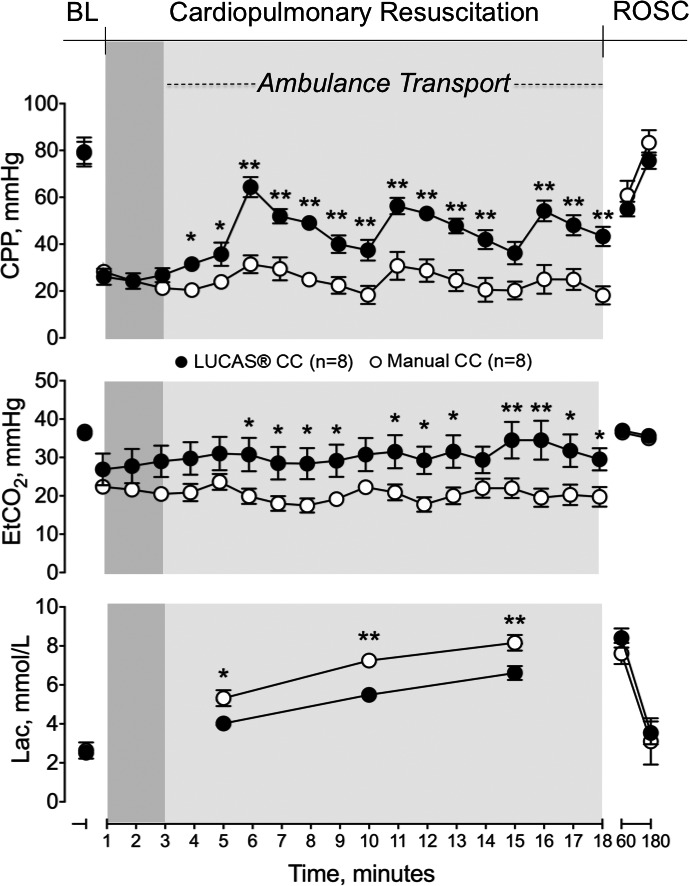
Coronary perfusion pressure, end tidal CO_2_, and arterial lactate levels (Lac) at baseline, during cardiopulmonary resuscitation, and after return of spontaneous circulation. BL indicates baseline; CPP, coronary perfusion pressure; EtCO_2_, end tidal CO_2_; Lac, arterial lactate levels; ROSC, return of spontaneous circulation. *P<0.05, ^†^ *P*<0.01 vs manual chest compression.

During the initial 3 minutes of CC, performed in the static condition, CPP was equivalent in the 2 groups. However, coincident with the onset of the ambulance movement and throughout the whole transport period, CPP was significantly higher in the mechanical CC group compared with the manual one (*P*<0.01, Figure 2).

**Figure 3:**
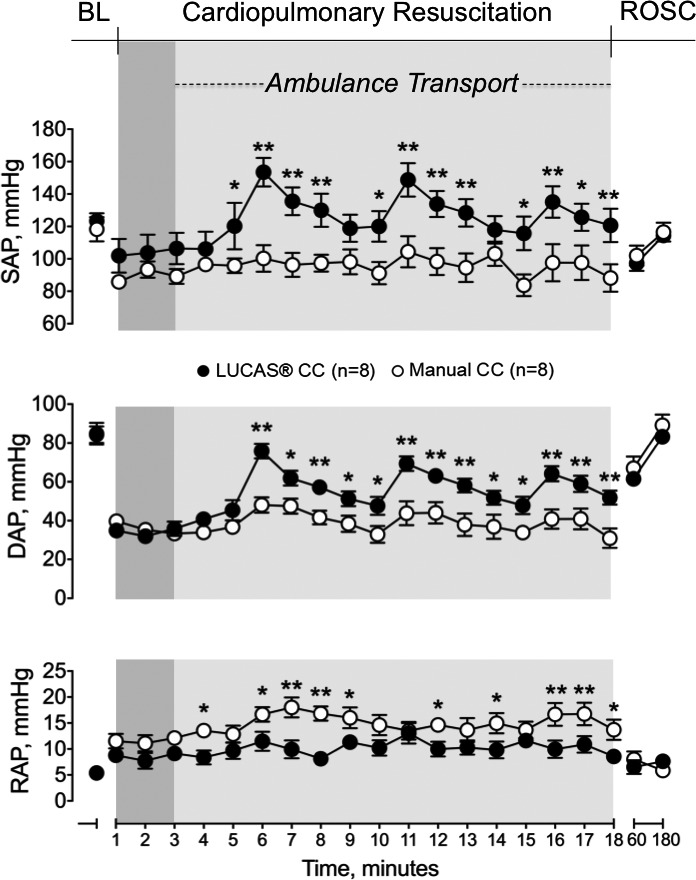
Systolic (SAP) and diastolic (DAP) arterial pressure, and right atrial pressure (RAP) at baseline, during cardiopulmonary resuscitation, and after return of spontaneous circulation. BL indicates baseline; DAP, diastolic arterial pressure; RAP, right atrial pressure; ROSC, return of spontaneous circulation; SAP, systolic arterial pressure. *P<0.05, ^†^ *P*<0.01 vs manual chest compression.

Similarly, EtCO_2_, systolic and diastolic arterial pressures were not different in the 2 groups during the static condition, while they were significantly higher in the mechanical CC compared with the manual one during ambulance transport (*P*<0.01, Figures 2 and 3). Right atrial pressure, instead, significantly increased in the manual CC group compared with the mechanical one during transport (*P*<0.01, Figure 3).

Arterial lactate showed a significantly greater increase in the manual CC group compared with mechanical one during the whole CPR period (*P*<0.01, Figure 2).

No differences in post‐resuscitation hemodynamics and arterial lactate were observed between the 2 groups (Figures 2 and 3).

### CPR Quality and Feasibility During Transport

Data on CPR quality and feasibility are summarized in Table 2.

CC rate was similar in the 2 groups during the overall duration of CPR and complied with current guidelines recommendations.^2^ Nevertheless, CC rate was constant at 102 per minute in the mechanical CC group, while it slightly varied in the manual one over time (*P*<0.01 versus LUCAS CC). The number of CCs delivered per minute and the CCF were overall significantly higher in the mechanical CC group compared with the manual one during ambulance transport.

**Figure 4:**
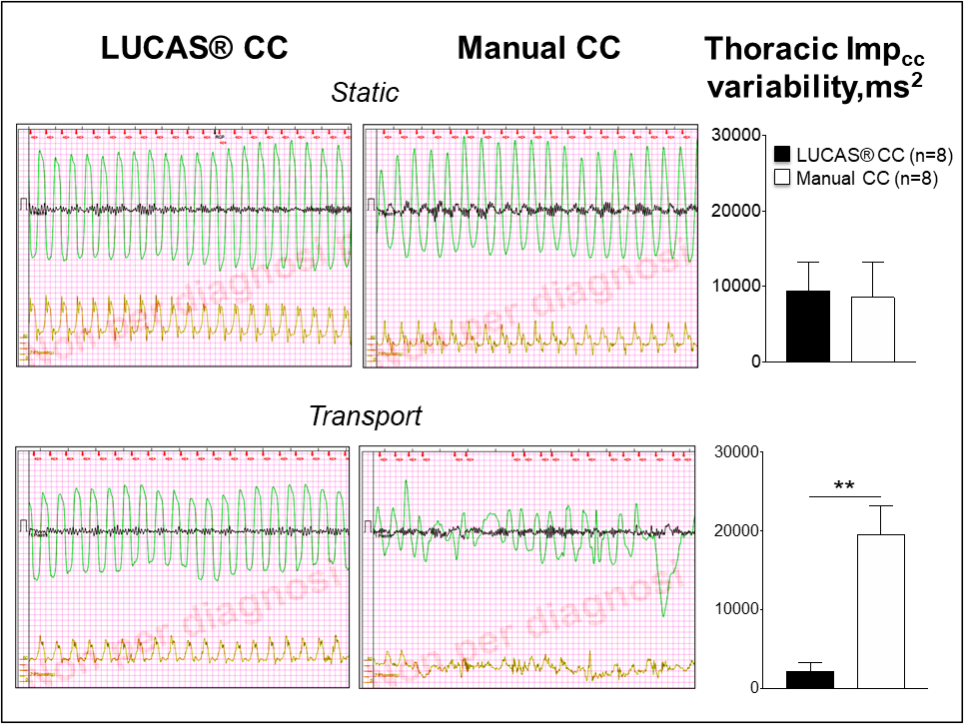
LUCAS (on the left) and manual (on the right) chest compression‐generated transthoracic impedance signal (in green) and corresponding arterial pressure (in orange) during cardiopulmonary resuscitation performed in static condition (on the top) and in the moving ambulance (on the bottom). The graphs on the right represent the CC‐generated transthoracic impedance variability in the LUCAS in the manual chest compression during the static condition (on the top) and the ambulance transport (on the bottom). CC indicates chest compression. *P<0.01 vs manual chest compression.

CC was more consistent in the mechanical CC group compared with the manual one during the whole period of CPR, as represented by a significantly lower variability in the CC‐generated TTI curve with the use of LUCAS 3 (*P*<0.01 versus manual CC, Table 2). More specifically, Imp_cc_ variability was similar between the 2 groups during the static condition, while it was >4‐fold greater in the manual CC group compared with the mechanical one during transport (*P*<0.01, Figure 4).

CPR providers described the manual CC during ambulance transport as significantly more physically exhausting (*P*<0.01) and less feasible (*P*<0.01) compared with the mechanical CC (Table 2).

### CPR Outcome and Survival

All 8 (100%) animals in the mechanical CC group and 6 (75%) in the manual one achieved ROSC (*P*=0.47, Table 3). Only a single defibrillation attempt was required before ROSC in the mechanical CC group compared with almost 2 in the manual one (*P*=0.06, Table 3).

**Table 3:** CPR Outcome

	LUCAS (n=8)	Manual (n=8)
ROSC, n (%)	8/8 (100)	6/8 (75)
Defibrillations to first ROSC, n	1±0	1.8±0.4
Defibrillations to final resuscitation, n	1.8±0.4	2.5±0.6
CPR duration, min	18±0	18.7±0.4
Rib fractures, n	5±1	5±1
72‐h survival, n (%)	7/8 (88)	6/6 (100)
72‐h OPC, score	1.5±0.5	1±0
HR, bpm		
PR 60 min	136±14	148±7
PR 120 min	125±14	114±8
PR 180 min	120±13	111±7
Temperature, °C		
ROSC	36.9±0.2	37.2±0.2
PR 60 min	36.4±0.3	36.6±0.4
PR 120 min	36.4±0.3	36.4±0.3
PR 180 min	36.4±0.3	36.3±0.3
CO, L/min		
PR 180 min	2.6±0.2	2.9±0.2
PR 72 h	4.1±0.4	3.6±0.5
EF, %		
PR 180 min	60±4	53±12
PR 72 h	76±2	77±2
EDV, mL		
PR 180 min	31±5	33±4
PR 72 h	38±5	37±2
ESV, mL		
PR 180 min	13±4	17±6
PR 72 h	9±2	9±1
hs‐cTnT, pg/mL		
PR 180 min	210 [91–619]	562 [381–687]
PR 72 h	38 [21–80]	41 [12–144]
72‐h NSE, ng/mL	0.18 [0.06–0.25]	0.16 [0.05–0.37]

Data are reported as mean±SEM, except for hs‐cTnT and NSE that are expressed as median [interquartile range]. CO indicates cardiac output; CPR, cardiopulmonary resuscitation; EDV, left ventricular end‐diastolic volume; EF, left ventricular ejection fraction; ESV, left ventricular end‐systolic volume; HR, heart rate; hs‐cTnT, high‐sensitivity cardiac troponin T; NSE, neuron‐specific enolase; OPC, overall performance category; PR, post resuscitation; ROSC, return of spontaneous circulation.

No differences in body temperature at ROSC and after resuscitation and in the total number of fractured ribs were observed between the 2 groups (Table 3).

All the resuscitated animals survived for 72 hours with a complete neurological recovery, except 1 in the mechanical CC group, which died 4 hours after resuscitation as a consequence of a hypertensive pneumothorax occurring during the transfer back to the cage (Table 3). No differences in post‐resuscitation arterial blood gases (Table 4), myocardial function, assessed by left ventricular ejection fraction and CO, and plasma levels of NSE and high‐sensitivity cardiac troponin T were observed between the 2 groups (Table 3). Nevertheless, in the early post‐resuscitation period, a consistently lower high‐sensitivity cardiac troponin T accompanied by a better ejection fraction and a lesser increased left ventricular end‐systolic volume was observed in animals subjected to mechanical CC compared with those that received manual CC (Table 3).

**Table 4:** Post‐Resuscitation Arterial Blood Gas Analyses

	LUCAS (n=8)	Manual (n=8)
pH		
PR 60 min	7.27±0.02	7.28±0.01
PR 180 min	7.40±0.01	7.41±0.02
PaO_2_, mmHg		
PR 60 min	99±12	126±6
PR 180 min	117±11	136±3
PaCO_2_, mmHg		
PR 60 min	44±2	44±3
PR 180 min	43±1	42±2
SpO_2_, %		
PR 60 min	94±2	98±0
PR 180 min	98±1	99±0
HCO_3_, mmol/L		
PR 60 min	20±1	20±1
PR 180 min	26±0	27±1
BE, mmol/L		
PR 60 min	−7±1	−6±1
PR 180 min	2±1	2±1

Data are reported as mean±SEM. BE indicates base excess; HCO_3_, bicarbonate; PaCO_2_; arterial carbon dioxide partial pressure; PaO_2_; arterial oxygen partial pressure; PR, post‐resuscitation; SpO_2_, arterial oxygen saturation.

## Discussion

To our knowledge, this is the first investigation describing and comparing the hemodynamic support generated by a piston‐based mechanical CC versus manual CC during ambulance transport in an experimental model of cardiac arrest and CPR. This randomized, animal study demonstrated that mechanical CC allowed for a significantly greater systemic perfusion during transport, as represented by higher CPP, EtCO_2_, arterial pressure, and better tissue oxygenation evident with lower arterial lactate, compared with manual CC. During ambulance transport, the use of a mechanical piston compression device also accounted for better CC quality, with a lesser rescuer's physical effort requirements, compared with manual compression.

Coronary perfusion pressure is the main determinant of myocardial blood flow and threshold levels of CPP have been identified as leading predictors of CPR success.^7^, ^10^, ^28^, ^29^ Indeed, maintaining a CPP >20 mmHg has been shown to increase the likelihood of ROSC and survival in both preclinical and clinical studies.^7^, ^29^, ^30^, ^31^ In the present study, a CPP ≈ 20 mmHg was achieved in the manual CC group, but >2‐fold greater values were observed during mechanical CC. CPP generated during CPR have been shown to be directly related to the quality of CC and more specifically to the depth.^10^, ^32^ In this study, the quality of compression, derived from the TTI signal was suboptimal in the manual CC compared with the mechanical one during transport. This might have been likely associated with provision of CC with shallow depth, as previously reported in manikin studies.^22^, ^33^ Moreover, right atrial pressure significantly increased during ambulance transport in the manual group compared with the mechanical one, accounting for the lower CPP. Higher right atrial pressure in the manual CC group might have been the consequence of the suboptimal CC quality provided, which produced low CO and forward blood flow. A possible rescuers’ leaning on the animal chest to warrant a stable position against the vehicle's movements might be another valid explanation.^22^, ^23^, ^34^

Similarly, capnography is another valuable tool to monitor the physiological effects of CPR, as it reflects pulmonary blood flow and indirectly the CC‐generated CO.^7^, ^10^, ^35^ During prolonged CPR, failure to achieve an EtCO_2_ >10‐15 mmHg has shown a strong correlation with unsuccessful resuscitation.^35^, ^36^, ^37^ In this study, EtCO_2_ achieved the above thresholds, nevertheless, it was consistently higher in the mechanical CC group compared with the manual one during transport, anticipating a greater effectiveness of CC delivered mechanically.^10^, ^38^, ^39^ Somewhat surprising, during the static condition no differences in CPP, EtCO_2_, and hemodynamics were detected between the 2 groups, indicating a manual CC of high quality, comparable with that of the mechanical piston device. During transport, however, the use of the LUCAS provided a constant and reliable CC performance, which resulted in a higher perfusion and lesser increase in arterial lactate. The sharper increases in arterial pressure after each epinephrine administration in the mechanical CC group in contrast to the blunted response in the manual CC provides additional evidence of the better hemodynamic support generated by mechanical CC during transport.^40^

Adequate CC rate and CCF during CPR have been demonstrated to be associated with greater likelihood of ROSC and survival after cardiac arrest.^2^, ^13^, ^14^ Thus, a CC rate between 100 and 120 per minute and a CCF of at least 60% have been recommended.^2^ In this study, the mechanical piston device worked constantly, with a CC rate consistently stable at 102 per minute, with no variance, both in static condition and during ambulance transport. In the manual group, the CC rate fully complied with current guidelines but showed a greater variance, similarly to what has been previously reported on manikins.^22^, ^23^, ^41^, ^42^ In 4 pigs, mechanical CC needed to be interrupted immediately after onset of CPR to allow for LUCAS repositioning on the chest and this explains the unexpected lower CCF compared with the manual CC group noted during the static condition. The high CC quality, in terms of CC rate and fraction, in the manual group was likely achieved because of the presence of the metronome guide provided by the defibrillator. With this feedback, rescuers were able to compress the chest with the correct rate, even under the difficult condition created by the moving ambulance.

Deterioration of the manual CC consistency during transport has been recognized to be strongly influenced by the ambulance movements.^23^ Indeed, it has been reported that sudden changes in the ambulance speed may increase vibrations and induce rescuer's unnecessary movements that potentially impact on CC depth and rate, and on forces applied on the patient's chest.^41^ Moreover, the transport‐generated external forces, ie, acceleration, deceleration, centrifugal forces in curves, have been shown to make manual CC physically more demanding and less effective.^22^ In a moving ambulance, maintaining the standard 2‐handed CC technique has been also reported to be not feasible for the majority of the transport time, since providers are usually forced to perform CC with 1 hand, and the other to support themselves.^43^ Average speed in our study was similar in both groups as was the ambulance itinerary. However, in the animals resuscitated manually, episodes of shallow CC, leaning, altered duty cycles and compression technique, 1‐handed CC, and not correct hands position on the chest, were present. In contrast, transport seemed to have no effects on mechanical device performance, which remained stable and independent from motion influences throughout the whole ambulance journey.

Furthermore, delivery of manual CC in a moving ambulance has been described as physically exhausting, not easily reproducible, and potentially unsafe by the CPR team involved in this study. Indeed, CC performed during transport by an unrestrained provider has been considered as a hazardous situation, potentially dangerous for both the provider and the patient, and for this the mechanical devices have been suggested since they may allow providers to remain seated and restrained while CC is delivered.^16^, ^23^, ^42^, ^44^ In our study, there were no injuries, however in several instances during the transport, the rescuer's stable position was compromised and falls or nearby falls occurred, worsening the CPR quality. Moreover, additional risks for the CC provider might come from the distraction attributable to focusing on CC, and unexpected movements of the ambulance.^22^ Besides providing consistent high‐quality CC, mechanical compression devices might therefore significantly reduce the above described risks and improve ambulance safety practices.

This study has several strengths. The investigation provides evidence of hemodynamics generated and maintained in a moving vehicle by both a mechanical piston device and manual CC. A great effort was done to reproduce a real clinical scenario of ambulance transport with ongoing CPR in an urban area, ie, a clinical ambulance with human medical equipment and professional rescuers were used. The study results add evidence to the current knowledge gap on mechanical CPR devices as claimed in the 2015 International Consensus on CPR.^16^

### Limitations

Some limitations deserve to be mentioned. The studies were conducted in healthy anesthetized animals and therefore in the absence of underlying diseases or injuries that are causative of cardiac arrest and with potential anesthesia‐related effects. Secondly, the time of untreated VF was relatively short, ie, 2 minutes, to be comparable with a real out‐of‐hospital cardiac arrest scenario and to account for a relevant myocardial ischemia.^45^ Nevertheless, the aim of the study was to investigate the hemodynamics during CPR in a moving ambulance, while effects on survival or long‐term outcome will be assessed in future studies using more clinically relevant durations of no‐flow.^46^ Thirdly, CC depth was not assessed, and thus the impact of transport on this CPR parameter can be only speculated based on the TTI signal and CPP. However, data on CC depth have been already reported in earlier studies performed on manikins, whereas no data on hemodynamics have been present yet. Fourthly, our rescuer team was well trained for the task requested by the experimental protocol and could optimize the CC performance based on the resulting arterial pressure and EtCO_2_ monitored on the defibrillators. Thus, it is likely that a CC with a quality superior than current standard has been delivered. Nevertheless, effects on hemodynamics were still not comparable with those from the LUCAS device during transport. Moreover, in accordance to what was reported in the clinical scenario,^43^ frequently CC providers had to perform 1‐handed CC, using the other hand to support themselves and prevent accidental falls because of transport‐generated external forces.^22^, ^41^ In these instances, the 1‐handed CC technique remains the only option to perform CC with minimal interruptions, in the absence of devices specifically designed to stabilize the provider in a moving ambulance.^47^ Accordingly, the efficacy of the 1‐handed CC technique compared with the standard 2‐handed approach needs future investigations. Finally, rescuers focused only on providing uninterrupted CC with no need for delivering bag ventilation because animals were mechanically ventilated. However, the use of a standardized mechanical ventilation in both groups allowed for an unbiased comparison of EtCO_2_ between the 2 CPR strategies.

## Conclusions

In this preclinical model of CPR performed in a moving ambulance, a piston‐based mechanical CC allowed for a significantly greater hemodynamic support and systemic perfusion, as represented by higher CPP, EtCO_2_, and arterial pressure, and lower arterial lactate, compared with manual CC. Mechanical CC accounted also for a better CC quality, with a lesser rescuer's physical effort requirements, compared with manual compression. This study provides evidence to suggest and encourage the use of mechanical devices during ambulance transport to assure ongoing high‐quality CC, tissue perfusion, and rescuers’ safety.

## Sources of Funding

The study was supported by equipment and grants from Stryker/Jolife AB, Lund, Sweden, which did not influence to any extent any of the data analysis.

## Disclosures

Dr Hardig is an employee of Stryker/Jolife AB. The remaining authors have no disclosures to report.
